# Recurrent Nipple Duct Obstruction in Two Breastfeeding Patients: A Case Report and Discussion of the Underlying Pathophysiology

**DOI:** 10.1007/s10911-025-09576-6

**Published:** 2025-02-01

**Authors:** Anna Sadovnikova, Susan Greenman, Bridget Young, Casey Rosen-Carole

**Affiliations:** 1 School of Medicine, University of California, Davis, Sacramento, CA USA; 2 Swedish Health Services, Seattle, WA USA; 3 Department of Pediatrics – Breastfeeding and Lactation Medicine, University of Rochester School of Medicine and Dentistry, Rochester, NY USA; 4 Departments of Obstetrics-Gynecology and Pediatrics – Breastfeeding and Lactation Medicine, University of Rochester School of Medicine and Dentistry, Rochester, NY USA

**Keywords:** Breastfeeding, Nipple pain, Breast pain, Breast pump, Clogged duct, White spot on nipple, Milk bleb, Lactolith

## Abstract

Nipple pain is a common reason for premature breastfeeding cessation. There exists anecdotal evidence that one cause of lactational nipple pain is a ductal obstruction, but there is no published literature describing this phenomenon. Herein we present two case reports for two patients who experienced breast and nipple pain concurrent with milk flow reduction. Both patients removed a small stone-like obstructing object from their nipple; this action was painful for one of the patients, resulting in immediate release of milk and relief from breast pain. Both patients experienced recurrence of stone formation in their nipple ducts. We analyzed the mineral composition of the obstructing objects and breast milk using inductively coupled mass spectroscopy. We use literature on teat obstructions in dairy cows and dacryolith and sialolith formation to propose hypotheses as to how the formation of obstructing objects in milk ducts might occur. Future research directions for determining the pathophysiology, clinical presentation, and management of human nipple duct obstructions are discussed.

## Introduction

Breast and nipple pain is a common lactational complaint [1]. Often, lactating patients present to their health provider with what they perceive as a painful obstruction of a milk duct [2–4]. A milk bleb, sometimes described as a “white spot,” is one type of nipple orifice obstruction, while a “blocked or clogged duct” is perceived by patients as a lump deep in the breast [2–5]. These “clogs” are reported to resolve with massage, breastfeeding, or milk expression; some patients describe a sudden return of milk flow from a previously obstructed area (i.e., the “clog”) with softening of that breast tissue after the removal or release of obstructing “stringy and soft” or “hard and spikey” material. Lactating individuals in online fora (Reddit and BabyCenter) have described a “grain of sand” or “shard” which, upon removal (sometimes painful) resulted in a spray of milk from the affected nipple duct and immediate relief from pain. There has not been scholarly or clinical literature describing this phenomenon.

Expert consensus suggests that breast softening described by patients is due to the release of milk sequestered in the alveolar lumen previously obstructed by edema, not by a “clog” of milk [5]. In fact, recent recommendations from the Academy of Breastfeeding Medicine discourage the use of massage and heat as these measures increase breast edema and the risk of breast abscess and phlegmon formation [5]. Likewise, nipple manipulation is discouraged as that may introduce infection or further traumatize the area. Yet, there is scant literature on the etiology of nipple orifice or ductal obstruction during lactation, so the diagnosis and treatment of these conditions remains a challenge. A literature search in NCBI PubMed using search terms “calculi”, “calculus”, “lactiferous”, “lacteal”, “mammary”, “breast”, “duct”, “teat”, “obstruction”, “lactolith”, “milk stone” and “blockage” revealed a number of publications on teat obstructions in dairy cows, but none in humans [6–8]. Teat obstructions in dairy cows are primarily due to the use of nonphysiologic vacuum suction on the teat end, resulting in its inflammation and eventual hyperkeratosis (i.e., scarring). There is a need to better characterize human nipple orifice obstruction to elucidate the pathophysiology, clinical presentation, and management of this subtype of lactational nipple and breast pain.

Here, we report two cases of nipple duct obstruction in two breastfeeding patients. The obstructing objects resembling a stone, shard, or grain of sand were removed by the patients, photographed, and subjected to mineral analysis by inductively coupled plasma (ICP)-mass spectrometry. We provide several hypotheses for the underlying pathophysiology of the formation of stones in the nipple ducts and suggestions for future research.

### Case 1

A 33-year-old primiparous breastfeeding patient (pronouns: they/them) presented at 2 months postpartum to our breastfeeding medicine clinic with nipple pain and a milk bleb. This patient had had a healthy, term singleton infant who was gaining appropriately, consuming primarily breastmilk with some formula supplementation. Previously, the patient presented for lactation support at 20 days postpartum for nipple pain, where the nipple was noted to be “lipstick shaped” and erythematous following breastfeeding. The infant was diagnosed with ankyloglossia and underwent frenotomy, which improved parental pain. At the time of their presentation to our breastfeeding medicine clinic at 2 months postpartum, the patient reported feeding at the breast 8 times per day and formula feeding by bottle 4 times daily (5 oz per day: 4 am, 7 am, 2 pm, 7 pm). They reported occasional breast pump use (Spectra, settings: 70/3, 54/4; output 12 ml) and had one 4 oz bag of frozen breast milk stored. The patient’s milk bleb was treated with nipple soaks and triamcinolone 0.1% twice daily with occlusive dressing, as was the standard management for milk blebs at the time, although saline nipple soaks are no longer recommended due to risk of tissue maceration [5]. One week later, the patient described “squeezing a 2 mm spheroid stone” from their nipple. Henceforth, the patient had recurrent breast engorgement, “clogged ducts,” nipple pain, and milk blebs which they treated with lecithin supplements (4800 mg daily), topical triamcinolone, hot compresses, massage, hand expression, and coconut oil soaks. These recommendations were made before the revised Academy of Breastfeeding Medicine protocol for management of the mastitis spectrum [5].

**Fig. 1:**
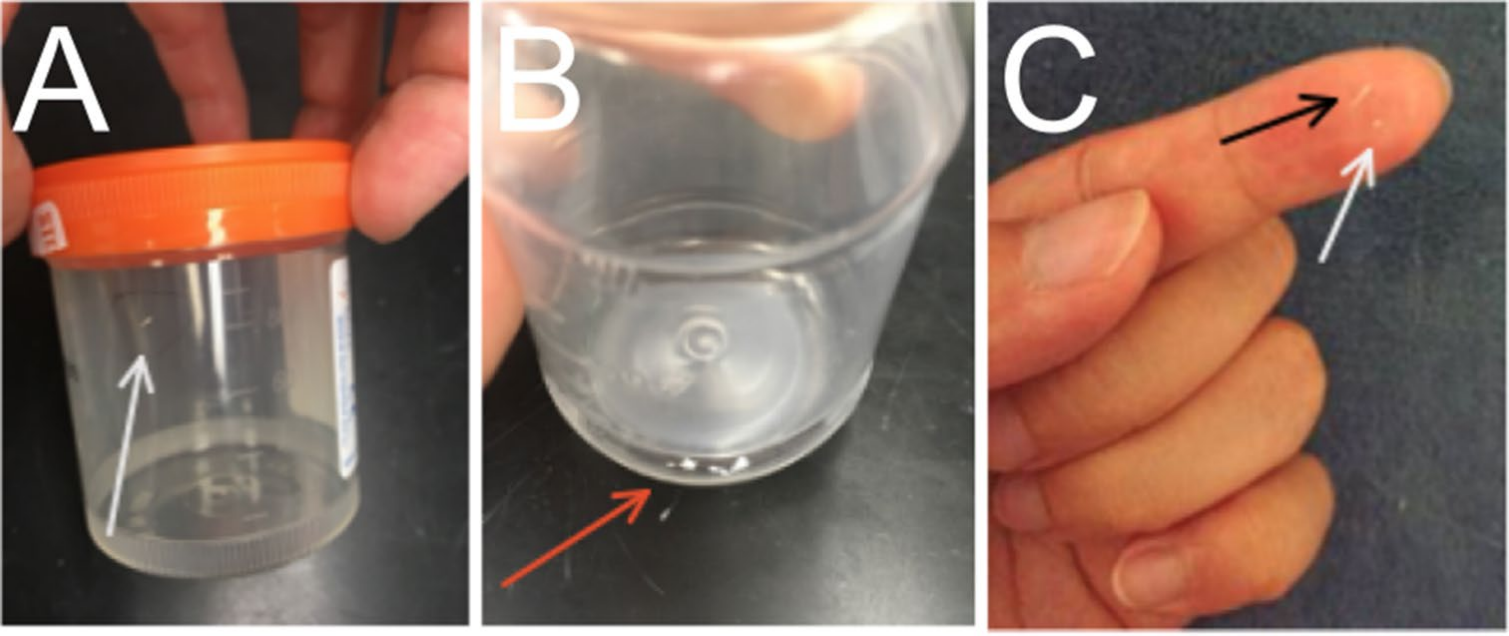
Obstructing objects from Case 1. **A**. A stone (arrow) from the patient’s left breast. Per the patient, stones from the left breast tended to be long and “spiky”. **B**. A series of 5 small, rounded stones (arrow) removed from the patient’s right breast. **C**. A comparison of left (black arrow) and right (white arrow) breast stones

The patient experienced breast pain every few weeks, usually following periods of difficult nursing reportedly associated with infant nasal congestion, breast engorgement, or inadequate parental water intake. To resolve the breast pain, they used their fingers “like squeezing a zit” or tweezers to remove between “2 and 6 shard-like, spikey slivers from the left nipple and round stones from the right” (Fig. 1).The patient continued to use tweezers despite recommendations against this practice. The patient reported that “stone” removal resulted in large amounts of breast milk spontaneously releasing from the previously engorged area of the breast and immediate relief from nipple and breast pain. The patient described the removal of the “stones” as “stunningly painful, and [taking] a long time to fully work up to the surface of the nipple.” Once it reached the nipple tip, the patient “could…feel through the nipple that it was finite, hard, round, and not the stringy thing that everyone kept talking about.”

Dietary changes did not significantly affect the incidence of ductal obstruction. This patient was taking oral iron supplements and escitalopram (10 mg) and averaged 630 mg/day of calcium intake (per a 7-day food diary). Her total serum Ca was 8.7 mg/dL. Elimination of dairy, soy and calcium rich foods from her diet did not affect the incidence of stone formation. The patient reported that an increase in hydration to at least 1.5 L per day reduced the incidence of nipple orifice blockage. The patient was lost to follow up. At their last contact at 15 months postpartum, it was discussed that if the patient continues to experience recurrent breast pain, they should obtain breast imaging (i.e., ultrasound and mammography) and may consider weaning the affected side. It is not known for how long they breastfed.

### Case 2

**Fig. 2:**
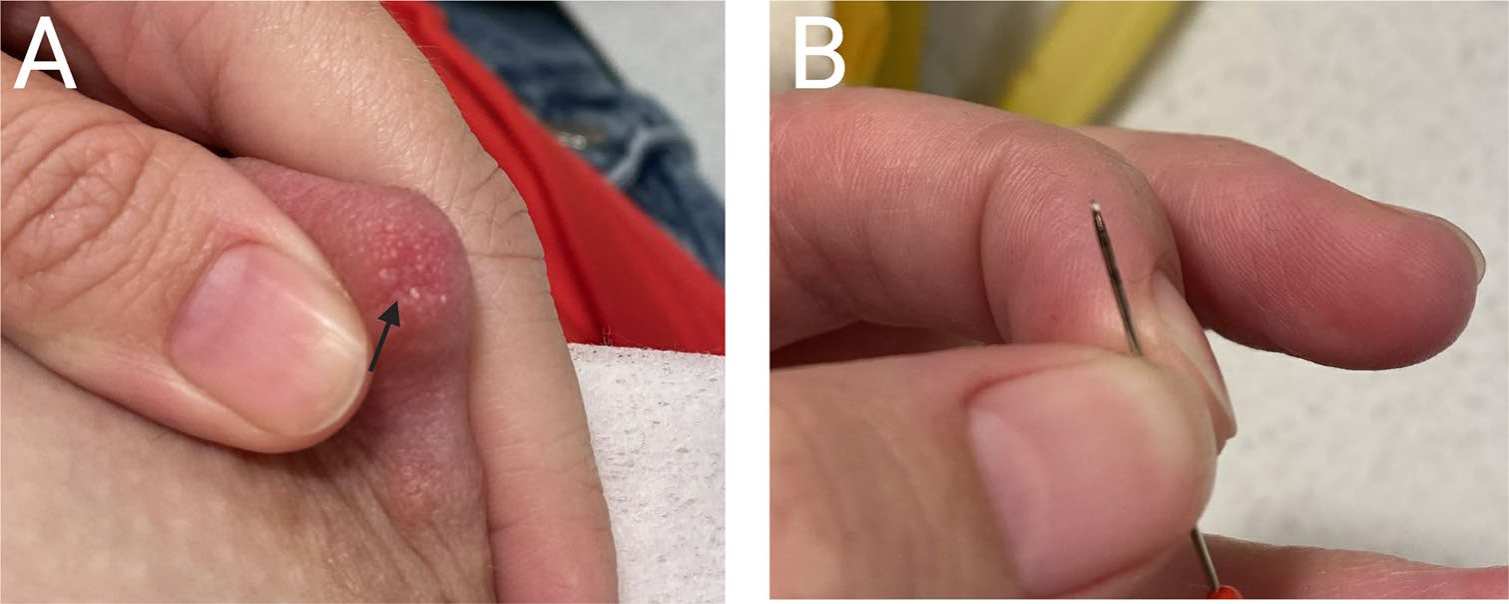
Obstructing objects from Case 2. **A**. Stones (arrows) are visible at the tip of the patient’s nipple. **B**. A stone is shown on the tip of a pin (arrow)

A 38-year-old multiparous breastfeeding parent (pronouns: she/her) of a term singleton infant presented at 2 months postpartum with a complaint of persistent “breast clogs.” The infant was exclusively breastfed and gaining weight appropriately. In the first month postpartum, the patient pumped only to provide a bottle of breastmilk on rare occasions. Some breastmilk (16 oz) was stored from previous pumping to preserve milk production during brief periods away from her baby. Since one month of age, the baby fed every 1–3 h during the day, slept overnight, and the mother did not pump. In the two weeks prior to presentation, the patient noted an increase in “clogged ducts” which seemed to originate from a blocked ductal opening. Though she reported not touching or massaging her breasts regularly, when she noticed a clogged duct and breast engorgement, she was able to extrude a hard “stone-like object” from the nipple tip, which immediately relieved the clogged duct. She reported that passing the stone was not painful, but the “clog” in the breast was painful. Figure 2 shows pictures of expressed objects held by the parent.

On evaluation, the infant had a dysfunctional suck and poor latch, which was not fully resolved after adjusting alignment and positioning. Ankyloglossia and an inflexible upper lip were diagnosed, but the family elected to defer frenotomy due to good milk transfer, adequate infant growth, and breast pain associated only with “stones.” The patient found that hydration decreased the occurrence of “stones,” though lecithin supplementation (4800 mg daily) did not. She initially reported weekly stones, then cessation of stones after her menses returned at 4 months postpartum. She experienced one more stone a few months later. She breastfed for 18 months.

## Methods

Patients removed the obstructing objects (stones) from their nipple and provided them to the study team. Expressed milk sample aliquots were also provided by patients from a full expression of a single breast. Milk macronutrient content was assessed via mid-infrared spectroscopy following sonication according to manufacturer’s instructions (Human Milk Analyzer; Miris, Uppsala, Sweden). Samples were analyzed in duplicate and the average of the results reported. True protein is reported. Milk and object elemental analyses were analyzed using a Perkin Elmer 2000 C ICP Mass Spectrometer, following digestion in concentrated (69%) trace metal grade nitric acid and heat treatment to 95 °C for one hour and dilution in ultra pure water (18 Megohm) to a total volume of 10 mL.

## Results

The breast milk macronutrient composition for Patient 2 (data not available from Patient 1) was more energy dense with higher fat and carbohydrate content than what has been reported in the literature for people producing mature milk (Table 1), suggesting that the breast milk sample provided by Patient 2 may have contained more hindmilk than foremilk [8]. For both patients, calcium and sulfur were the two most abundant minerals found in the objects (Table 2). Yet, there was a significant difference (*p* = .039) in the relative abundance of the top ten most common elements found within each object between patients. In particular, the object in Case 2 had zinc and silicon as the third and seventh, respectively most abundant minerals, whereas for Case 1, zinc and silicon were 11th and 12th, respectively, in abundance (Table 2). By contrast, there was no difference (*p* = .19) in the relative abundance of the six most common elements in the milk samples from the two patients (*p* = .195) (Table 3).

**Table 1:** Breast milk composition

Sample	Fat (g/100mL)	Carbohydrates (g/100mL)	Energy (kcal/100mL)	Protein (g/100mL)
Patient 2	6.4	8.7	100.0	1.00
Control	3.5-4.0	6–7	65–70	0.8-1.0

Macronutrient and energy composition from Case 2 are compared to average mature breast milk macronutrient and energy content

**Table 2:** Stone element composition presented in order of abundance

Element	Case 1 (PPM)	Element	Case 2 (PPM)
Ca	8583	S	24,895
S	6861	Ca	9867
P	2854	Zn	5000
Na	1727	Na	4959
K	976	Fe	3112
Mg	274	K	3095
Ti	166	Si	2564
Fe	86	P	1998
Al	63	Mg	858
Cl	61	Al	692

The top ten most abundant analytes in each stone from Cases 1 and 2 are presented. (PPM = parts per million)

**Table 3:** Breast milk mineral composition. Common breast milk minerals presented in alphabetical order

Element	Case 1 (PPM)	Case 2 (PPM)
Ca	37	52
K	435	150
Mg	37	29
Na	118	114
P	142	121
S	13	7

PPM = parts per million

## Discussion

This is the first case report to describe breast and nipple pain caused by a hard, removable object obstructing the nipple orifice in two lactating people. We suspect that there is a spectrum of disease presentation: milk blebs and stones lie on a continuum. Repeat trauma from the breast pump, infant latch, or manual manipulation results in a pathological inflammatory response at ductal opening that triggers a cascade of events eventually leading to fibrosis with or without stone formation. While the patient in case 1 engaged in some significant nipple manipulation, the patient in case 2 did not, suggesting manipulation is not the only source of this particular breast pathology. Nevertheless, patients should be advised against manual manipulation of white spots on their nipple to avoid additional trauma and worsening of their condition. Milk blebs treatment has been outlined elsewhere [2–5]. Both patients had a similar clinical presentation to those described in the online fora consisting of nipple trauma from latch and/or breast pump use, hyperlactation, and nipple and breast pain relieved after stone removal. Likewise, both of our patients and several patients in online fora have described not being able to remove the stones without painful manipulation, suggesting that the advice to not manipulate the breast may be hard to follow.

Breast calcifications are known to occur throughout the lifespan and may represent benign or cancerous lesions in the breast, but these calcifications are predominantly composed of calcium oxalate and calcium phosphate, with mineralogical differences between malignant and benign lesions poorly understood [9]. The Ca:P, Mg:Ca, and Na:Ca ratios of the stones were different from those reported for breast calcifications [9], suggesting breast calcification is unlikely to be the etiology for the obstructing objects experienced by our patients.

Objects obstructing milk ducts, called “lactoliths,” have been described in the veterinary medicine and dairy science literature, but their composition and etiology has not been defined [10, 11]. What has been described are proximal and internal teat canal obstructions, which are thought to contribute to stone formation. There is, of course, a difference in the diameter and length between the cow teat canal and human nipple ducts, which limits our ability to translate what is known about stone formation in the cow teat canal to the human experience. A major anatomical difference between the teat and the nipple is that, by definition, a teat only has one galactophore (milk duct) while the nipple has two or more. The diameter of all galactophores is smallest at its orifice, progressively increasing until it reaches the cistern (in cows) or the nipple base (in humans).

**Fig. 3:**
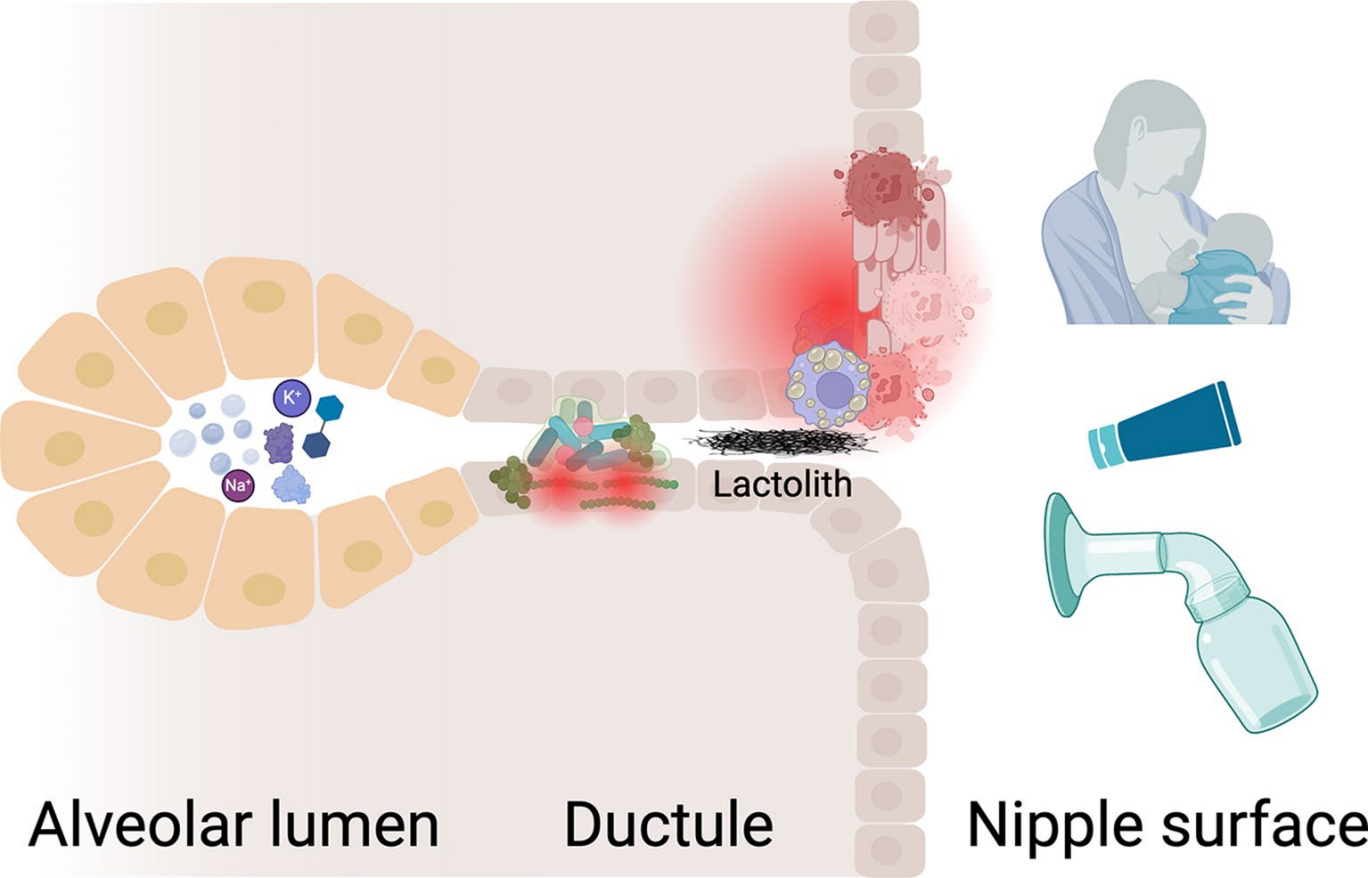
Proposed mechanisms of ductal stone formation. The primary insult leading to stone formation is likely due to external trauma (**A**) at the ductal orifice. In humans, there are numerous causes of nipple apex trauma, from a latch problem to breast pump use or topical agents that cause microtrauma, inflammation, and thickening of the skin at the ductal orifice (**B**). Internal ductule trauma or malformation (*not shown in figure)* likely co-exists with injury at the orifice. In dairy cows, teat hyperkeratosis damages the anti-microbial barrier, resulting in microbial dysbiosis (**C**) in the teat canal. Likewise, high vacuum, especially when “dry milking” before stimulating a let down, is associated with mucosal avulsion within the teat canal, and can lead to stricture of the teat canal. Finally, some individuals may be prone to stone formation based on the composition of their breast milk, milk microbiota, or diet (**D**). While less likely, it is possible that some individuals have a congenital anomaly in their milk ducts (*not shown in figure)* such that there is an increased likelihood of milk stasis with or without the translocation of microbes from the skin surface, resulting in stone formation

Since the mammary gland is a tubulo-acinar exocrine gland that shares many similarities with other glandular structures, we also sought to find examples in other exocrine glands of ductal obstructions where the duct is comparable in diameter and length to the human nipple. We found numerous reports of painful obstructions of lacrimal (dacryoliths) and salivary (sialoliths) ducts and glandular structures, which, akin to the mammary gland, secrete a fluid that then traverses a long, small duct before expulsion. Using the literature on teat obstructions in dairy cows and dacryolith and sialolith formation as a framework, we have developed three potential mechanisms by which an obstructing object in the human nipple duct may form (Fig. 3): (1) nipple orifice irritation, trauma, or obstruction, (2) nipple duct inflammation, fibrosis, stricture, or other anatomic defect, and (3) factors deep within the mammary tree that promote stone formation in the human nipple duct.

### External Trauma at the Ductal Orifice

**Fig. 4:**
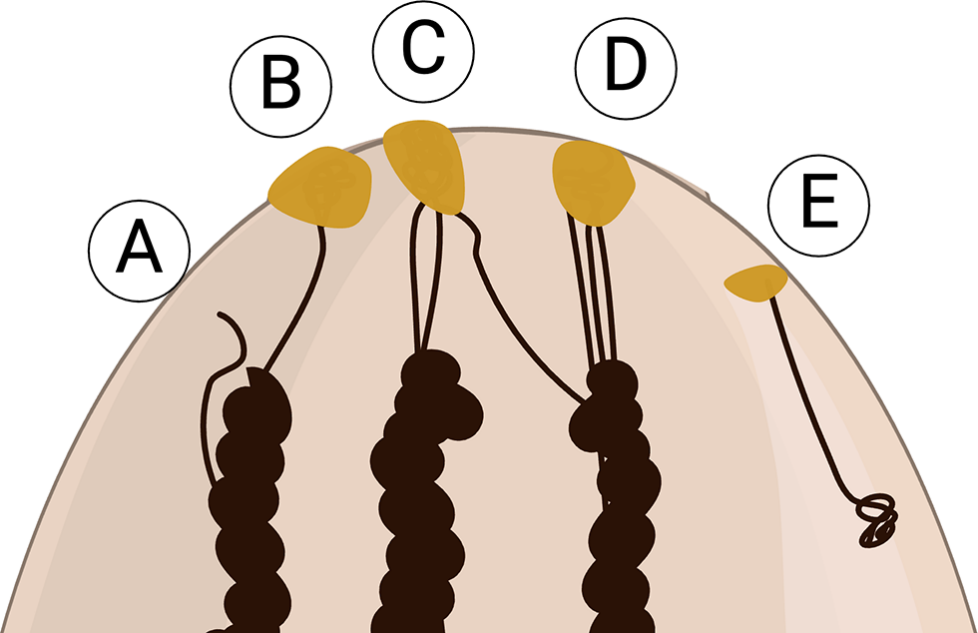
A simplified cartoon showcasing variations in the microanatomy of ducts in the human nipple. Larger ducts from the mammary gland come together at a “waist” at the level of the nipple base. On the nipple apex, there are numerous crevices where one or more ductal openings exist. As the ducts travel through the nipple towards its apex, they can end in a blind pouch (**A**), exit the nipple through an orifice as a single ductal opening (**B**) or with other ductules (**C** and **D**), or, potentially, change routes and leave their “bundle” to join a different group at an orifice (**D** to **C**), although this last situation is less likely based on what is known about mammary embryology. Likewise, there are other ducts and sebaceous glands that are not milk-secreting located in the nipple (**E**). Adapted from Rusby et al. [12]

We believe that external trauma to the ductal orifice is an important factor in the development of an obstructing object in the duct. Both of the patients in this case series had grossly intact nipples on physical exam, but had experienced nipple trauma from latching an infant with ankyloglossia. We cannot exclude the possibility that nipple skin was additionally irritated by manipulation with forceps during stone removal (Case 1), breast pump use, new topical agents, nursing pads, or bras that were used in the postpartum period, resulting in inflammation of the skin at the nipple orifice (Fig. 3). Consistent with this latter concern was the presentation of Case 1 with a nipple “bleb,” typically thought to represent hyperkeratotic obstruction of an inflamed nipple duct orifice [4]. Teat obstruction in dairy cows is almost always trauma-related (> 90%) and not due to a congenital anatomic defect in the teat canal itself [10, 11]. External factors, including high vacuum from the milk claw and topical irritants, are known to cause fibrous remodeling, epithelial metaplasia, and loss of specialized blood vessels resulting in complete scarring over of a teat opening (teat hyperkeratosis, akin to a milk “bleb” in humans) or nonfunctioning downstream segments of a nasolacrimal ductule, respectively. Human nipples are especially prone to external insults during the lactation experience, as single, small ducts branch and coalesce multiple times within the human nipple, unlike in dairy cows (Fig. 4) [12]. Several tiny ductules open to one orifice at the nipple apex (Fig. 4), or one ductule leaves a cluster of ducts to join a new one at a different orifice [12]. Therefore, if one ductal orifice is blocked, there can be deleterious downstream effects on numerous, seemingly unpredictable ducts and segments of the mammary gland.

### Internal Ductule Trauma or Malformation

The presence of a blockage at the level of the ductal orifice may not directly translate to the formation of a stone in the nipple duct. It is likely that a secondary hit (e.g., two-hit hypothesis) is required, such as damage to internal ductal structures, although, in cows, severe teat hyperkeratosis is thought to be associated with the development of mastitis in that quarter [13]. The teat canal, and possibly the nipple duct, can also be damaged by the vacuum if it is turned on before milk let down occurs. If there is no milk flowing from the udder to the teat canal, as would occur after let-down, the walls of the teat canal collapse, thereby subjecting the fragile epithelium lining the teat canal to further damage, resulting in mucosal avulsion with the possibility of hematoma formation; repeated injury can result in teat canal stricture and loss of that udder quarter [13]. The same may be true for women using the breast pump frequently, not allowing their breast tissue to recover in between pumping sessions, resulting in repeated microtrauma within nipple ductules, which can present as bloody milk with or without blood clots. Once the inflammatory or constricted environment is created, an obstructing object in the duct could begin to form.

A congenital abnormality in the ductule itself may also be responsible for promoting an stone-forming environment. In the example of the dacryolith, its composition was strongly correlated to whether it was formed in the lacrimal sac versus the canaliculus, where concretions devoid of cells (i.e., non-infectious origins) were found exclusively within the lacrimal sac [14]. There is also evidence that an abnormality in a valve of Hasner–a fold of the mucous membrane at the end of a nasolacrimal duct that prevents air from entering the lacrimal sac from the nose–is associated with dacryolithiasis [14]. It is possible that the patients in our case report had something unusual about the anatomy of their milk duct–its circumference, length, tortuosity–that increased the likelihood of stone formation.

### Milk Composition and Microbiota

The third and final ingredient in the recipe of stone formation is likely the milk composition and microbial community. In recent years, it was discovered that the innermost core of the sialolith is composed of bacteria [15]. It was hypothesized that the biofilm produced by bacteria (e.g., *Streptococcus* spp.) attracts calcium carbonates and calcium phosphates from the saliva, resulting in mineralization and concretion formation [15]. It is well-known that breast milk and nipple skin contains a multitude of bacterial and fungal organisms, which vary based on a number of maternal characteristics [16, 17]. The same is true in the dairy cows, for whom we have additional evidence that there are three distinct bacterial communities in the teat end apex, teat canal, and milk [18, 19]; it is not known whether the same differential occurs in human mammary tree. Milk acts as a potent selective medium that precludes the growth of specific bacterial lineages; once there is teat apex inflammation, internal canal stricture or trauma, or a change in the milk composition, the microbial communities are perturbed and the result is often mastitis and, possibly for some individuals, stone formation. As we highlighted earlier, the dairy cows’ environment and teat anatomy differs from that of the human, yet they remain superior to many other animal models for hypothesis generation of how microbial communities might impact human nipple stone formation. Their utility has become especially salient as they (alongside goats and a few other livestock dairy animals) are the only other mammals besides humans for whom vacuum pumps are used to regularly remove milk, thereby serving as an elegant animal model for the effects of the breast pump on nipples/teats, milk composition and volume, and animal health.

It is possible that our patients’ breast milk composition primed stone formation (Fig. 3). For example, saliva, urine, and tear composition, respectively, can affect lithiasis. Patients with and without frequent dacryoliths have differences in tear composition, where those with chronic dacryoliths have lower lysozyme and potassium levels, but similar calcium levels to healthy controls [14, 20]. It is thought that low lysozyme levels reduce proteolysis and may create a permissive environment for lithiasis. Sialoliths are composed of proteins at their core and surrounded by inorganic molecules. In one study, lysozyme (95%), lactoferrin (85%) and secretory immunoglobulin A (s-IgA) (75%) were found in nearly all of the submandibular sialoliths [21]. Given that lactoferrin, lysozyme, and s-IgA are also found in high concentrations in breast milk, it would be important to understand how breast milk composition affects stone composition, a patient’s predisposition for stone formation, and the associated symptomatology.

### Parental Diet and Water Intake

Diet has been shown to increase the risk of subclincial and clinical mastitis in humans and dairy cows due to the creation of a pro-inflammatory milieu [22–25]. Our patient in case 1 experimented with elimination of various foods, tracking their intake with a food diary, and measuring serum Ca. At this time, we do not have sufficient evidence to implicate maternal diet in the formation of stones. Our patients reported an improvement in symptoms with adequate water intake. While adequate water intake is important for prevention of nephrolithiasis, it is not known whether the pathophysiology of nephrolithiasis is similar to that of stones in the milk ducts [26]. In the kidney, stones form by a process called nucleation, whereby initial crystal seeds form when ions/molecules cluster together in the nephron or renal calyces. Nucleation of kidney stones is secondary to an altered urine pH, concentration of stone-forming substances, and presence/absence of inhibitory compounds—this process is similar in concept to what we have proposed for stone formation in the nipple duct. Both of the patients in this case report described an inverse relationship between water intake and stone formation, but prospective clinical trials are needed to better understand the relationship between maternal diet and water intake and the milk bleb-stone spectrum of conditions.

### Limitations

Several important limitations should be considered when interpreting these findings. Most important is that with only two patients, our ability to generalize these findings is limited. While we performed mineral analysis on the stones, we were unable to conduct comprehensive structural analysis, microscopy, or bacterial culture or DNA sequencing of the specimens. Likewise, complete milk samples and compositional analyses were only available from one patient, limiting comparative analyses. We cannot fully exclude the role of mental health factors, including obsessive-compulsive behaviors, that may have contributed to recurrence through repeated manipulation and trauma of the nipple tissue, especially in Case 1. Finally, our long-term follow-up was limited, as one patient was lost to follow-up and the other experienced only one additional occurrence before weaning at 18 months, preventing us from fully understanding the natural history of stone formation and its impact on breastfeeding outcomes.

### Recommendations for Future Research

We recommend a prospective case series of patients presenting with obstructing objects in their nipple ducts. If a patient has recurrent stone formation, it would be ideal to collect the data described below for each event, and determine inter- and intra-patient variability of nipple anatomy, micro/mycobiome, and stone composition. We do not recommend ductoscopy, as this is an invasive procedure that has low sensitivity and specificity given the small diameter of ductules at the nipple orifice and the numerous pathways a ductule can take from the nipple apex to base [12].

1. **Complete lactation history**, with aspecial focus on signs and symptoms of nipple trauma, including:

- breastfeeding practices, pump use.
- topical and oral medication or agents, type of bra and nursing pads or other items in contact with nipple skin.
- infant anatomy and latch characteristics.
- Mental health history, including obsessive-compulsive behaviors.

2) **Imaging**, including:

- Dermatoscopic image of the nipple apex.
- Non-invasive optical biopsy of the nipple orifice to define the histology of the nipple epidermis surrounding the ductal orifice with reflectance confocal microscopy [27], optical coherence tomography, quantitative oblique back illumination microscopy [28], or similar non-invasive optical biopsy method.

3) **Stone characterization and analysis**, including:

- Macroscopic/magnified photography of the removed stone.
- Composition analysis of the stone, including mineral, protein, carbohydrate, lipids.

4) **Microbial analysis**, including:

- 16s rRNA and ITS sequencing of the microbial communities of stone, milk, and nipple skin using minimally-invasive techniques such as microprojection arrays and swabs. Tissue stripping may be painful [29].
- Bacterial and fungal culture of milk and nipple skin.

## Conclusion

This is the first case report describing the clinical presentation and mineral composition of stones in two breastfeeding patients’ nipples. Stone formation is likely influenced by trauma at the nipple skin surface contributing to inflammation at the ductal orifice, dysbiosis and inflammation inside the ductule, and alterations in the milk composition. Future case reports should include comprehensive history-taking, skin dermoscopy and optical biopsy, stone microscopy and compositional analyses, skin and milk microbiome evaluation, and comprehensive breast milk composition analyses.

## Data Availability

No datasets were generated or analysed during the current study.

## References

[1] Berens P, Eglash A, Malloy M, Steube AM. ABM Clinical Protocol #26: Persistent Pain with Breastfeeding. Breastfeed Med. 2016;11(2):46–53. 26881962 10.1089/bfm.2016.29002.pjb

[2] McGuire E. Case study: White spot and lecithin. Breastfeed Rev. 2020;23(1):23–5. 25906494

[3] Sadovnikova A, Fine J, Tartar DM. Differences in diagnosis and Treatment of Nipple Conditions of Reproductive-Age Women at a Tertiary Health System. J Womens Health (Larchmt). 2023;32(12):1388–93. 37917916 10.1089/jwh.2023.0231 PMC10712359

[4] Mitchell KB, Johnson HM. Breast Pathology that contributes to dysfunction of human lactation: a spotlight on Nipple blebs. J Mammary Gland Biol Neoplasia. 2020;25(2):79–83. 32495215 10.1007/s10911-020-09450-7

[5] Mitchell KB, Johnson HM, Rodríguez JM, Eglash A, Scherzinger C, Zakarija-Grkovic I, et al. Academy of Breastfeeding Medicine Clinical Protocol #36: the Mastitis Spectrum, revised 2022. Breastfeed Med. 2022;17(5):360–76. 35576513 10.1089/bfm.2022.29207.kbm

[6] Sahoo S, Ganguly S. Successful management of lactolith (milk stone) in a Red Sindhi Cow-A case study. Int J Contemp Pathol (Institute Medico-legal Publications New Delhi India). 2016;2:47–8.

[7] Franz S, Floek M, Hofmann-Parisot M. Ultrasonography of the bovine udder and teat. Veterinary Clin North America: Food Anim Pract. 2009;25(3):669–85. 10.1016/j.cvfa.2009.07.007 19825439

[8] Abd-El-Hady A. Clinical observations on some surgical udder and teat affections in cattle and buffaloes. In 2015 [cited 2024 May 23]. Available from: https://www.semanticscholar.org/paper/Clinical-observations-on-some-surgical-udder-and-in-Abd-El-Hady/15b0c1ecb62a067904693cfe1a2151727e47ab77

[9] Scott R, Kendall C, Stone N, Rogers K. Elemental vs. phase composition of breast calcifications. Sci Rep. 2017;7:136. 28273938 10.1038/s41598-017-00183-y PMC5427875

[10] Seeh C, Melle T, Medl M, Hospes R. [Systematic classification of milk flow obstruction in cattle using endoscopic findings with special consideration of hidden teat injuries]. Tierarztl Prax Ausg G Grosstiere Nutztiere. 1998;26(4):174–86. 9710918

[11] Ducharme NG, Arighi M, Horney FD, Livesey MA, Hurtig MH, Pennock P. Invasive teat surgery in dairy cattle: part I - surgical procedures and classification of lesions. Can Vet J. 1987;28(12):757–62. 17422937 PMC1680567

[12] Rusby JE, Brachtel EF, Michaelson JS, Koerner FC, Smith BL. Breast duct anatomy in the human nipple: three-dimensional patterns and clinical implications. Breast Cancer Res Treat. 2007;106(2):171–9. 17221150 10.1007/s10549-006-9487-2

[13] Odorčić M, Rasmussen MD, Paulrud CO, Bruckmaier RM, Review. Milking machine settings, teat condition and milking efficiency in dairy cows. Animal. 2019;13(S1):s94–9. 31280747 10.1017/S1751731119000417

[14] Mishra K, Hu KY, Kamal S, Andron A, Della Rocca RC, Ali MJ, et al. Dacryolithiasis: Rev Ophthalmic Plast Reconstr Surg. 2017;33(2):83. 10.1097/IOP.0000000000000769 27533513

[15] De Grandi R, Capaccio P, Bidossi A, Bottagisio M, Drago L, Torretta S, et al. Salivary calculi microbiota: new insights into microbial networks and pathogens reservoir. Microbes Infect. 2019;21(2):109–12. 30385304 10.1016/j.micinf.2018.10.002

[16] Boix-Amorós A, Puente-Sánchez F, du Toit E, Linderborg KM, Zhang Y, Yang B, et al. Mycobiome profiles in breast milk from healthy women depend on Mode of Delivery, Geographic Location, and Interaction with Bacteria. Appl Environ Microbiol. 2019;85(9):e02994–18. 30824446 10.1128/AEM.02994-18 PMC6495746

[17] Zimmermann P, Curtis N. Breast milk microbiota: a review of the factors that influence composition. J Infect. 2020;81(1):17–47. 32035939 10.1016/j.jinf.2020.01.023

[18] Dean CJ, Slizovskiy IB, Crone KK, Pfennig AX, Heins BJ, Caixeta LS, et al. Investigating the cow skin and teat canal microbiomes of the bovine udder using different sampling and sequencing approaches. J Dairy Sci. 2021;104(1):644–61. 33131828 10.3168/jds.2020-18277

[19] Derakhshani H, Plaizier JC, De Buck J, Barkema HW, Khafipour E. Composition and co-occurrence patterns of the microbiota of different niches of the bovine mammary gland: potential associations with mastitis susceptibility, udder inflammation, and teat-end hyperkeratosis. Anim Microbiome. 2020;2(1):11. 33499931 10.1186/s42523-020-00028-6 PMC7807822

[20] Lew H, Lee SY, Yun YS. Measurement of pH, electrolytes and electrophoretic studies of tear proteins in tears of patients with dacryoliths: a novel concept for dacryoliths. Ophthalmologica. 2004;218(2):130–5. 15004503 10.1159/000076149

[21] Kraaij S, de Visscher JGAM, Apperloo RC, Nazmi K, Bikker FJ, Brand HS. Lactoferrin and the development of salivary stones: a pilot study. Biometals. 2023;36(3):657–65. 36396778 10.1007/s10534-022-00465-7 PMC10181970

[22] Gay MCL, Koleva PT, Slupsky CM, Toit E, du, Eggesbo M, Johnson CC, et al. Worldwide Variation in human milk metabolome: indicators of breast physiology and maternal lifestyle? Nutrients. 2018;10(9):1151. 30420587 10.3390/nu10091151 PMC6163258

[23] Afeiche MC, Iroz A, Thielecke F, De Castro AC, Lefebvre G, Draper CF, et al. The Dietary Inflammatory Index is Associated with subclinical mastitis in Lactating European Women. Nutrients. 2022;14(22):4719. 36432405 10.3390/nu14224719 PMC9696022

[24] Zeng Y, Zhang D, Fu N, Zhao W, Huang Q, Cui J, et al. Risk factors for granulomatous mastitis and Establishment and Validation of a clinical prediction model (Nomogram). Risk Manag Healthc Policy. 2023;16:2209–22. 37881167 10.2147/RMHP.S431228 PMC10596285

[25] Wang L, Wang Y, Meng M, Ma N, Wei G, Huo R, et al. High-concentrate diet elevates histone lactylation mediated by p300/CBP through the upregulation of lactic acid and induces an inflammatory response in mammary gland of dairy cows. Microb Pathog. 2023;180:106135. 37172660 10.1016/j.micpath.2023.106135

[26] Bao Y, Tu X, Wei Q. Water for preventing urinary stones. Cochrane Database Syst Rev. 2020;2020(2):CD004292. 10.1002/14651858.CD004292.pub4 PMC7012319 32045491

[27] Cinotti E, Galluccio D, Tognetti L, Habougit C, Manganoni AM, Venturini M, et al. Nipple and areola lesions: review of dermoscopy and reflectance confocal microscopy features. J Eur Acad Dermatol Venereol. 2019;33(10):1837–46. 31166040 10.1111/jdv.15727

[28] Abraham TM, Costa PC, Filan C, Guang Z, Zhang Z, Neill S, et al. Label- and slide-free tissue histology using 3D epi-mode quantitative phase imaging and virtual hematoxylin and eosin staining. Optica OPTICA. 2023;10(12):1605–18. 39640229 10.1364/optica.502859 PMC11620277

[29] Liang K, Leong C, Loh JM, Chan N, Lim L, Lam YI, et al. A 3D-printed transepidermal microprojection array for human skin microbiome sampling. Proc Natl Acad Sci U S A. 2022;119(30):e2203556119. 35867832 10.1073/pnas.2203556119 PMC9335308

